# RE: The value of testicular ultrasound in the prediction of the type and size of testicular tumors

**DOI:** 10.1590/S1677-5538.IBJU.2015.0728

**Published:** 2016

**Authors:** Abraham Shtricker, David Silver, Elias Sorin, Letizia Schreiber, Nachum Katlowitz, Alexander Tsivian, Kalman Katlowitz, Shalva Benjamin, Abraham Ami Sidi

**Affiliations:** 1 Department of Urologic Surgery, Edith Wolfson Medical Center, Sackler school of medicine, University of Tel Aviv, Israel; 2 Maimonidis Medical Center-NY - Department of Urologic Surgery, New York, NY, USA; 3 Department of Radiology, Edith Wolfson Medical Center, Sackler school of medicine, University of Tel Aviv, Israel; 4 Department of Pathology, Edith Wolfson Medical Center, Sackler school of medicine, University of Tel Aviv, Israel; 5 Staten Island University Hospital-NY - Department of Urologic Surgery, New York, NY, USA

*To the editor,*

We read with great interest the article “The value of testicular ultrasound in the prediction of the type and size of testicular tumors” by Shtricker A. et al (1). This article highlights an interesting role of testicular ultrasound finding in managing testicular tumor, particularly regarding the ability of testicular ultrasound to differentiate between these three lesions: benign lesion, seminomatous germ cell tumor (SGCT) and non-seminomatous germ tumor (NSGCT). The presence of necrosis is more suggestive of malignant tumors, whereas hypoechogeneity and fibrosis on testicular ultrasound are more suggestive of SGCT type (1). These finding will increase the ability to differentiate type of testicular tumor preoperatively in addition to traditionally use tumor markers. Hopefully in near future the characteristic of different type of cancers can be done to form risk stratification Table.

On the other hand, this article gives us a big doubt regarding the ability of preoperative ultrasound to estimate the tumor size as compared to pathological measurement. It was not verified the actual time interval between diagnostic ultrasound and the orchiectomy. Fast growing cancer will give significant change in size within short period of time. Thus, the ultrasound findings will be smaller in comparison to pathological size if the time interval between the ultrasound and operation was a week or more. In contrast to malignant lesion, the benign lesion was well documented as slow growing and expected to have similar size during diagnostic ultrasound and pathological specimen regardless the time interval; as shown in this study that 100% of benign tumors showed similar sizes for both measurements (1, 2). Furthermore, the study was conducted in multicentre which will give more varieties in term of technique as ultrasound requires a highly experienced and skilled operator, as well as advance equipment (3). Besides that, in current practice only in cases of SGCT the tumor size will be taking into account for risk stratified prognosis (2). The other more important factor was histological features which determine the prognosisof both SGCT and NSGCT, however cannot be provided by ultrasound (2, 3). Thus, we strongly suggest strict protocol should be applied in organ sparing-surgery for non-tumour contralateral testis cases.

***Mohd Nazli Kamarulzaman, MD*** *Urology Unit, Department of Surgery* *International islamic University Malaysia* *Jalan Hospital Campus Jalan Penjara,* *Kuantan, Malaysia* ***Siti Kamariah Che Mohamed, MD*** *Department of Radiology* *International Islamic University* *Malaysia Kulliyyah of Medicine* *Kuantan, Pahang, Malaysia* *E-mail: nazlikamarulzaman@gmail.com*
